# Tunable Magneto-Plasmonic Nanosensor for Sensitive Detection of Foodborne Pathogens

**DOI:** 10.3390/bios13010109

**Published:** 2023-01-07

**Authors:** Tuhina Banerjee, Nilamben Panchal, Carissa Sutton, Rebekah Elliott, Truptiben Patel, Kajal Kajal, Eniola Arogunyo, Neelima Koti, Santimukul Santra

**Affiliations:** 1 Department of Chemistry and Biochemistry, College of Natural and Applied Sciences, Missouri State University, 901 S. National Avenue, Springfield, MO 65897, USA; 2 Department of Chemistry, College and Arts and Sciences, Pittsburg State University, 1701 S. Broadway Street, Pittsburg, KS 66762, USA

**Keywords:** magneto-plasmonic nanosensor, colorimetric detection, *E. coli* O157:H7, magnetic relaxation, surface plasmon resonance

## Abstract

Frequent outbreaks of food-borne pathogens, particularly *E. coli* O157:H7, continue to impact human health and the agricultural economy tremendously. The required cell count for this pathogenic strain of *E. coli* O157:H7 is relatively low and hence it is vital to detect at low colony forming unit (CFU) counts. Available detection methods, though sensitive, fall short in terms of timeliness and often require extensive sample processing. To overcome these limitations, we propose a novel magneto-plasmonic nanosensor (MPnS) by integrating surface plasmon resonance (SPR) properties with spin–spin magnetic relaxation (T2 MR) technology. We engineered MPnS by encapsulating several gold nanoparticles (GNPs) within the polymer-coating of iron oxide nanoparticles (IONPs). First, the polyacrylic acid (PAA)-coated IONPs were synthesized using a solvent precipitation method, then gold chloride solution was used to synthesize GNPs and encapsulate them within the PAA-coatings of IONPs in one step. A magnetic separation technique was used to purify the MPnS and the presence of GNPs within IONPs was characterized using transmission electron microscopy (TEM), energy dispersive x-ray spectroscopy (EDS), and other spectroscopic methods. The synthesized MPnS exhibits MR relaxation properties while possessing amplified optical properties than conventional GNPs. This allows for rapid and ultrasensitive detection of *E. coli* O157:H7 by SPR, T2 MR, and colorimetric readout. Experiments conducted in simple buffer and in milk as a complex media demonstrated that our MPnS-based assay could detect as low as 10 CFUs of this pathogenic strain of *E. coli* O157:H7 in minutes with no cross-reactivity. Overall, the formulated MPnS is robust and holds great potential for the ultrasensitive detection of *E. coli* O157:H7 in a simple and timely fashion. Moreover, this platform is highly customizable and can be used for the detection of other foodborne pathogens.

## 1. Introduction

*Escherichia coli* (*E. coli*) is a global public health risk and increasing economic burden responsible for foodborne outbreaks leading to severe illnesses and food recalls [1,2,3]. Infection rates have steadily risen since *E. coli* was first linked to human infection in 1982 and traced to undercooked meat [4]. Serotype O157:H7 has since been identified as the most lethal strain, Shiga toxin-producing *E. coli* (STEC) [1]. In 2005, the World Health Organization (WHO) reported 1.8 million deaths due to food contamination by pathogens, including *E. coli* serotype O157:H7 [5]. By 2014, an estimated 2.8 million global infections annually were attributed to STEC according to a systematic review by Majowicz et al. [6]. The Centers for Disease Control and Prevention (CDC) estimates an annual 48 million foodborne illnesses, 128,000 hospitalizations, and 3000 deaths in the US alone [7]. Of those cases, *E. coli* O157:H7 contributed to food contamination leading to 20 deaths, 2100 hospitalizations, and 63,000 infections [8]. Furthermore, the WHO documented STEC as the cause of 1 million illnesses, 100 deaths, and 13,000 disability-adjusted life years (DALY) [9]. *E. coli* O157:H7 is one of the most prevalent illness-provoking pathogens accounting for nearly 70 percent of foodborne transmission [10]. Successful prevention strategies such as the detection in agrifood are urgently needed.

Various factors, however, contribute to unsuccessful infection prevention and safety protocol implementation. *E. coli* O157:H7 contamination has been traced to numerous sources including dehydrated milk products with survivability also demonstrated in infant formula powders [11,12], leafy vegetables [13,14], and meat [15,16]. Food-packaging materials [17] and biofilm contamination [18] are also causes of concern. Transmission of infection can additionally be acquired by person-to-person contact [19], animal contact [20,21], airborne routes [22,23], and waterborne routes [24,25,26] including contaminated swimming pools [27]. *E. coli* O157:H7 has also demonstrated the ability to form biofilms and cause cross-contamination of products [18]. Further contributing factors to the rapid spread are the low infection dose of STEC of only 10–100 organisms [28]. In light of STEC’s high virulence and prevalence, even more alarming is that STEC strains are now demonstrating antibiotic resistance [29,30,31,32,33,34], with some isolates also reported to be multidrug resistant [35]. Rapid, specific, and sensitive testing is paramount in the agrifood industry to stymie this escalating worldwide public health risk.

Numerous testing methods have been developed to answer this need. Current FDA detection protocols require the traditional microbiological testing method, quantitative polymerase chain reaction (qPCR), which is dependent on whole genome sequencing (WGS) a single isolate [36]. A 24-h sample enrichment is needed followed by detection of the main virulence genes and O157 antigen by qPCR with a total analysis duration requiring two to three days with positive results leading to two to four additional days of testing [36]. Metagenomics is currently being explored, including 16S rRNA and shotgun metagenomic WGS. However, specificity is problematic using the 16S rRNA fragment which cannot easily distinguish between species-level identities [37], while conversely, shotgun metagenomic WGS is more specific but less sensitive with a detection limit above 103 CFU/mL [38,39,40,41]. While rapid detection methods have been sought to overcome these limitations, high costs and skilled technical staff have limited advancements [42]. Low sensitivity, false-negative results, and prolonged results due to a necessary pre-enrichment step to achieve sufficient cell surface antigens have limited immunological-based detection assays [43]. Biosensor-based methods have been shown to overcome most of the aforementioned obstacles but can require long incubation times and have demonstrated low specificity; hence, rapid and cost-effective detection has remained elusive [44,45].

Over the past few years, multi-functional nanomaterials have offered an exciting avenue for sensing applications. In particular, magneto-plasmonic nanomaterials [46,47,48,49,50,51] have been extensively used for biosensing applications for different target analytes. The superior detection sensitivity of these nanomaterials is attributed to their excellent surface chemistry, enhanced superparamagnetic properties, and unique optical properties made possible due to the integration of magnetic and plasmonic properties within a confined nanoscale area. Several conventional approaches [52,53,54,55] are known for the preparation of these unique materials, which involve, for example, the preparation of an iron oxide core followed by coating with gold. However, challenges associated with these synthetic protocols, including achieving dispersed formulations and getting uniform gold shell coatings, limit their application in attaining enhanced sensitivity and reproducibility for detection.

In this study, we demonstrate a new facile approach to fabricating a multimodal magneto-plasmonic nanosensor (MPnS) and its potential application in biosensing using *E. coli* O157:H7 as a target pathogen. Our MPnS synthetic approach involves two distinct steps: (1) the polyacrylic acid (PAA)-coated iron oxide nanoparticles (IONPs) preparation [56], and (2) in situ formation of gold nanoparticles (GNPs) within the PAA coatings of IONPs using the Turkevich method [57]. The proposed new synthetic strategy leverages the tunable plasmonic properties of GNPs determined by adjusting the PAA-polymer-coating thickness to achieve maximum sensitivity. In addition, MPnS displayed robust nanozyme properties and was applied for the development of sensitive detection platforms including ELISA and lateral flow assay (LFA) [58]. Notably, each fabricated MPnS contains several encapsulated GNPs with unique optical properties that exhibit size-dependent color changes on aggregation induced by the addition of a target, and hence offer amplified colorimetric detection.

To develop an ultrasensitive detection assay for *E. coli* O157:H7, MPnS are functionalized with specific antibodies for the target pathogen on the surface of nanosensors using NHS/EDC chemistry [59]. The MPnS-based detection assay uses a spin–spin MR mechanism based on the clustering of specific pathogens on the antibody-conjugated MPnS, which replace the neighboring water molecules from the Fe_3_O_4_ cores. This phenomenon results in observable changes in the spin–spin relaxation times of surrounding water protons (T2 MR) that are sensitively collected by a bench-top magnetic relaxometer. Importantly, bacterial concentrations as low as 10 colony-forming units (CFUs) were detected within minutes and were compared with available related methods (Table S1) [60,61,62,63,64,65,66]. Furthermore, added SPR- and colorimetric-detection modalities allow improved sensitivity and amplified visual detection, as demonstrated by marked differences in color and a large red shift in the SPR peaks. Additionally, in the presence of other bacterial targets, little to no interaction was observed demonstrating excellent specificity. However, one can use a magnetic column to further separate functional MPnS from non-specific analytes before measuring magnetic relaxation of detection. Comparing the analytical performance of the MPnS-based detection assay in simple and complex media indicated similar sensitivity. Overall, results suggest that our MPnS design provides a robust sensing platform for achieving ultrasensitive detection and at the same time equally applicable in field settings. Moreover, this novel technology can be readily adapted for other pathogens and help address urgent needs of global proportions for pathogen detection in the agrifood industry.

## 2. Experimental Section

### 2.1. Reagents

Hydrogen tetrachloroaurate (III) trihydrate (HAuCl_4_ × 3H_2_O) was procured from STREM chemicals, Inc. (Newburyport, MA, USA). Ferric chloride hexahydrate (FeCl_3_ × 6H_2_O), ferrous chloride tetrahydrate (FeCl_2_ × 4H_2_O), hydrochloric acid (HCl), ammonium hydroxide (NH_4_OH), phosphate buffer saline (PBS), and sodium citrate were acquired from Fisher Scientific (Waltham, MA, USA) and used as received. 2-Morpholinoethanesulfonic acid (MES) and polyacrylic acid (PAA) were purchased from Sigma-Aldrich (St. Louis, MO, USA). EDC (1-ethyl-3-[3-(dimethylamino) propyl] carbodiimide hydrochloride) was obtained from Pierce Biotechnology. N-hydroxy succinimide (NHS) was acquired from ACROS organics. The dialysis bag (MWCO 6–8 K) was purchased from Spectrum Labs. Bacterial strains *E. coli* O157:H7, salmonella typhimurium, and generic *E. coli* O111 were purchased from American Type Culture Collection (ATCC). *Anti-E. coli* O157:H7 antibody was obtained from Abcam.

### 2.2. Instrumentations

Spin–spin magnetic relaxation (T2 ms) studies were performed by using Bruker’s magnetic relaxometer mq20 (0.47 T, B = 20 MHz). Size, as well as zeta potential, of IONPs, GNPs, and MPnS, were measured using Malvern’s Zetasizer-ZS90. Tecan i-control infinite M200 plate reader was used for the measurement of surface plasmon resonance. Type-1 DI water (18.2 MΩ × cm at 25 °C) was obtained using GenPure UV-TOC, purchased from Thermo Scientific.

### 2.3. Bacterial Culture

Freeze-dried pellets of *E. coli* O157:H7 were hydrated with 5 mL of nutrient broth media. Subsequently, 100 µL of resuspended pellet was spread onto the agar plate using an L-shaped glass spreader and incubated for 24 h at 37 °C. Isolated colonies were picked and inoculated into 35 mL of nutrient broth and then incubated overnight at 37 °C with 200 rpm agitation. After overnight incubation, bacterial solution was centrifuged at 5500× *g* for 10 min. The pellet was then washed with PBS (1X, 7.4 pH) twice. Next, the optical density of the solution was adjusted to 0.1 and serial dilution was performed. Plate counting method was used for confirmation of colony-forming units (CFUs).

### 2.4. Synthesis of Gold Nanoparticles (GNPs)

To synthesize GNPs, 5 mM HAuCl4 (2.0 mL) (12 mg in 12.5 mL in DI water) was added into DI water (17 mL) at 95 °C in 50 mL Erlenmeyer flask placed on a hot plate with magnetic stir bar. After boiling for 10 min, 0.5% NaCit (1.0 mL) (5 mg in 1 mL DI water) was added to reaction mixture, with continued stirring and heating (Scheme S1A). The reaction was allowed to continue for 20 min until a ruby-red color was observed. The resulting solution was purified by dialysis (MWCO: 6–8 K) in water to remove unreacted chemicals. GNPs were stored at 4 °C. The GNPs’ average diameter and zeta potential were found to be 18 ± 2 nm and −26 mV, respectively, and they were also characterized using UV-Vis and magnetic relaxometer (Figures S1 and S2).

### 2.5. Synthesis of Iron Oxide Nanoparticles (IONPs)

The IONPs are synthesized using the water precipitation method, as our previously reported method [59]. Briefly, first, 3 solutions were made. Solution 1 contained FeCl_3_ (0.622 g), FeCl_2_ (0.334 g), and H_2_O (2 mL). Solution 2 contained NH_4_OH (1.8 mL of 30% stock) and H_2_O (15 mL). The third solution contained polyacrylic acid (0.859 g) and H_2_O (5 mL). After preparing all the solutions, HCl (90 uL, 12M) was added to solution 1 and then was immediately added to solution 2 in vortexing conditions at 800 RPM. Then, solution 3 was added to solution 2 in vortexing conditions with continued mixing for 1 h (Scheme S1B). After mixing, the solution was centrifuged at 2880× *g* to remove the larger particles. The supernatant was collected and dialyzed (molecular weight cutoff: 6–8 K) in DI water overnight to remove unreacted mixture. The purified IONPs ([Fe] = 4 mM) were stored at room temperature and characterized (Figures S1 and S2).

### 2.6. Synthesis of Magneto-Plasmonic Nanosensors (MPnS)

**Scheme 1:**
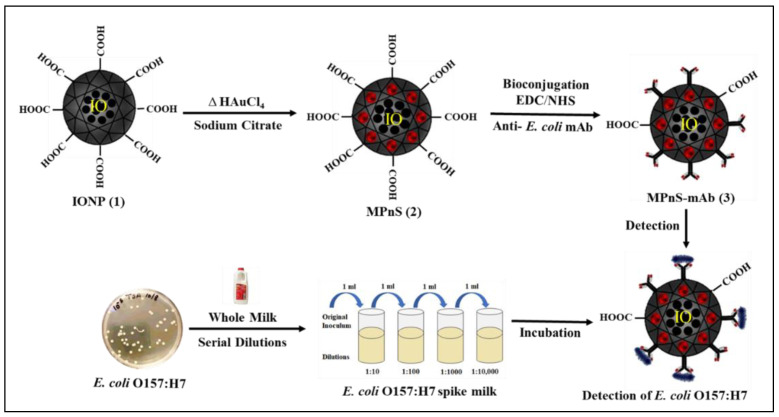
Schematic illustration of synthesis of magneto-plasmonic nanosensor (MPnS) and its conjugate for the multiparametric detection of *E. coli* O157:H7.

**Figure 1:**
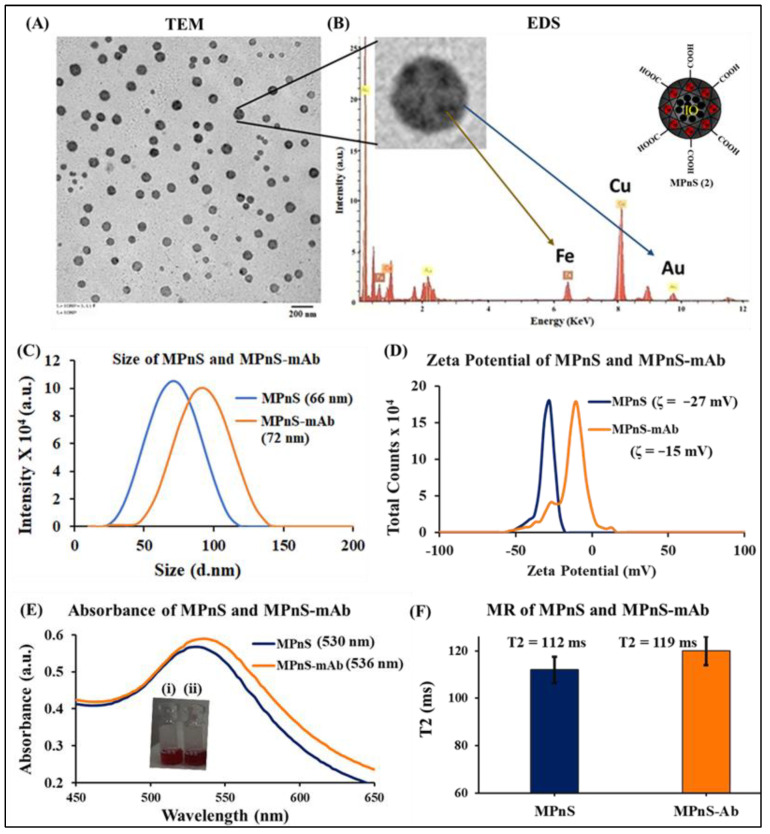
Characterization studies of MPnS and MPnS-mAb conjugates: (**A**) Transmission electron microscopy (TEM) image of MPnS, scale bar: 200 nm. (**B**) Energy-dispersive X-ray spectroscopy (EDS) of MPnS, showing the presence of bimetallic system. (**C**) Plot of hydrodynamic diameter. (**D**) Zeta potential of MPnS before and after surface conjugation. (**E**) UV-Vis absorption spectra of MPnS and the conjugate, confirming the successful antibody conjugation, inset: images of (i) MPnS and (ii) MPnS conjugate solution. (**F**) T2 values before and after surface modification of MPnS.2.7. Conjugation of target-specific antibodies to the surface of MPnS.

To synthesize MPnS, 5 mM HAuCl_4_ (2.0 mL) (12 mg in 12.5 mL in DI water) was added to IONPs (17.0 mL, T2 = 100–110 ms) in 50 mL Erlenmeyer flask (Scheme 1). The flask was placed on a hot plate and stirred at 500 RPM at 95 °C. After boiling for 10 mins, 0.5% NaCit (1.0 mL) (5 mg in 1 mL DI water) was added to the reaction mixture with continuous stirring and heating. Reaction continued for 20 min until wine-red color was observed. The MPnS ([Fe] = 2.0 mM) was purified by magnetic column and stored at 4 °C. The average diameter of these MPnS was found to be 68 ± 2 nm and detailed characterization results are shown in Figure 1.

For the conjugation of *anti-E. coli* O157:H7 IgG1 antibody on the surface of MPnS, to begin, four solutions were prepared. Solution 1 contained 5.0 mL of MPnS ([Fe] = 2.0 mM); solution 2 contained 8 µg of monoclonal *anti-E. coli* antibody in 1.0 mL of 1X PBS (pH 7.4); solution 3 contained 3 mg of N-hydroxysuccinimide (NHS) in 100 µL of MES buffer (pH = 6.0); and solution 4 comprised 5 mg of 1-ethyl-3-(3-dimethylaminopropyl) carbodiimide hydrochloride (EDC) in 100 µL of MES buffer. First, 100 µL of solution 4 was added to solution 1 and gently mixed for 10-–15 sec, followed by addition of 100 µL of solution 3 mixed for 3–4 mins. Then, antibody solution was added in small increments with 1 min intervals, and finally, the reaction mixture was placed on a tabletop mixer for 3 h at room temperature and then overnight at 4 °C. The conjugated MPnS-Ab solution was purified using a magnetic column to remove unconjugated antibodies and stored at 4 °C. The functional MPnS-Ab ([Fe] = 1.0 mM) was characterized by measuring the change in size, zeta potential, magnetic relaxation, and absorbance maximum, as shown in Figure 1.

### 2.7. Procedure for MPnS-Based Detection Assay

Following supplier’s bacteria culturing protocol, serial dilutions and plate counting were performed to determine CFUs and a dynamic detection range of 1.0–105 CFUs or higher was determined. In a typical detection assay, 200 µL of *anti-E. coli* O157:H7 IgG1-MPnS (1.0 mM) was incubated for 30 min at 25 °C with increasing CFUs of *E. coli* O157:H7 and corresponding MR data were collected on a Bruker’s relaxometer (0.47 T). Next, samples were placed in 96-well plate for UV-Vis measurements (SPR) and color transitions for each sample were recorded.

### 2.8. Specificity and Cross-Reactivity Detection Studies

Cross-reactivity studies were performed to determine the robustness and specificity of functional MPnS to the *E. coli* O157:H7. Pathogens such as Salmonella typhimurium (S. typhimurium) as well as isotypic control *E. coli* strain, *E. coli* O111, were assessed with *E. coli* O157:H7 as a standard. A mix of all pathogens was also evaluated. To each well containing the respective pathogens mixed in PBS, 200 µL of *anti-E. coli* O157:H7 IgG1-MPnS (1.0 mM) was added. A control solution with only *anti-E. coli* O157:H7 IgG1-MPnS was used.

### 2.9. Evaluation of Assay Performance in Complex Matrices

To validate the detection sensitivity of MPnS-Ab in complex media, detection studies and cross-reactivity tests were conducted in 10% milk diluted in 1X PBS (pH 7.4).

### 2.10. Procedure for MPnS Saturation Assay

In a 96-well plate, free *anti-E. coli* O157:H7 IgG1 (10 µM) was added to the different solutions containing increasing bacterial CFUs. Then, 200 µL of MPnS-Ab (1.0 mM) was added to each well. A control solution with only MPnS-Ab and no spiked bacteria was also added. Further, surface plasmon resonance (SPR) and magnetic relaxation (MR) studies were performed to confirm the specificity of interactions.

## 3. Results and Discussion

### 3.1. Working Principle of Functional MPnS-Ab for Detection of E. coli O157:H7

Using MPnS-Ab, target pathogens may be detected within minutes and without the need for sample amplification, which is required by many detection methods used today. Scheme 1 illustrates the concept of our sensing platform for sensitive and rapid detection of *E. coli* O157:H7. Integrated magnetic relaxation technology and surface plasmon resonance through the formulation of a magneto-plasmonic nanosensor (MPnS) allow qualitative and quantitative detection of the target pathogen. For the preparation of MPnS, a new synthetic protocol was designed that allowed the encapsulation of several GNPs on the PAA polymeric coating of IONPs. Cooperative interactions between different nanoparticles are expected to enhance SPR and MR signals compared to conventional iron oxide or gold nanoparticles. Since each MPnS contains several encapsulated GNPs, colorimetry-based detection is anticipated to achieve higher sensitivity. The successful conjugation of MPnS with the *E. coli* O157:H7 specific antibody further allows specific binding between the pathogen and the functional MPnS-Ab, resulting in detectable T2 MR and observable shift in wavelength maximum (ΔSPR) along with a visual read-out. With the increase in target concentrations, the interaction with MPnS-Ab further rises, leading to more clustering. This in turn displaces the water molecules around the MPnS-Ab accompanied by increased T2 MR values as monitored by the benchtop magnetic relaxometer. Likewise, a significant shift in ΔSPR arising from encapsulated GNPs is also observed. The SPR peak maxima shift is attributed to a change in the aggregation state of encapsulated GNPs that is quantitatively determined by UV-Vis measurements. Moreover, a change in SPR wavelength, if large enough, also produces an observable color change that makes our detection assay equally applicable for field-based settings. With the MPnS-based assay, quantitative bacterial concentrations as low as 10 CFUs were detected via T2 MR/SPR signals and a visual read-out of 100 CFUs was achieved. Additionally, our assay is sensitive enough to discriminate bacterial targets other than *E. coli* O157:H7.

### 3.2. Synthesis, Functionalization, and Characterization of MPnS

In the present study, a water-based synthetic method was adopted for constructing this iron-gold hybrid nanomaterial. The MPnS design involves two steps: (1) polyacrylic acid (PAA)-coated IONPs preparation, and (2) in situ formation of GNPs within the PAA coatings of IONPs using the Turkevich method [57]. The PAA-coated IONPs are synthesized using our previously reported protocol [59] and characterized using DLS and zeta potential measurements (Figure S1). The hydrodynamic diameter and surface charge of IONPs were found to be 55 ± 3 nm and −30 ± 2 mV, respectively. The superparamagnetic property of IONPs was confirmed by the collection of spin–spin T2 relaxation data (T2 = 89 ms). Prior to MPnS synthesis, citrate-coated GNPs preparation was standardized by varying the ratios of sodium citrate and gold chloride (HAuCl_4_) concentrations. DLS and UV-Vis measurements of synthesized GNPs under optimized conditions indicated a stable monodisperse formulation with a size of 18 ± 2 nm and a surface charge of −26 ± 2 mV with an SPR peak at 520 nm (Figure S1). Following the optimization of IONPs and GNPs syntheses, MPnS fabrication was conducted using the nanoprecipitation approach. The one-step GNPs synthesis and encapsulation within the polymeric coating of IONPs was carried out by mixing IONPs with gold chloride solution and followed by reduction at 100 °C. Reaction completion was marked by the appearance of a wine-red color (Figure S2), with a characteristic SPR peak at 530 nm (Figure 1). The transmission electron microscopic (TEM) image of MPnS showed the formation of monodispersed, IO-Au nanocomposite (scale bar 200 nm), and the energy dispersive X-ray spectroscopy (EDS) elemental analysis confirmed the presence of a bimetallic system (Figure 1A,B). The average diameter of MPnS was further verified by DLS and was found to be 66 ± 2 nm. The presence of the polyacrylic coating on MPnS was further confirmed by a corresponding negative zeta potential of −27 ± 2 mV (Figure 1C,D). The red shift of SPR peaks (from 520 nm for GNPs to 530 nm for MPnS) is indicative of the successful entrapment of GNPs in the PAA coatings of IONPs. Superparamagnetic properties of MPnS were verified by the collection of spin–spin T2 relaxation times and were found to be 112 ms (Figure 1E,F). Batch-to-batch variability of MPnS determined by measuring size, T2, and absorption maximum showed minimum variations [58]. Overall, these observations demonstrate the successful fabrication of a stable MPnS that has plasmonic, colorimetric, and magnetic relaxation properties. MPnS was then conjugated with specific monoclonal antibodies of *E. coli* O157:H7 (mAb) using NHS/EDC chemistry. Dynamic light scattering (DLS) was used to measure the change in overall size to demonstrate successful conjugation. The hydrodynamic diameter of the MPnS-mAb resulted in an expected increase from 66 ± 2 nm to 72 ± 1 nm, indicating successful conjugation with mAb. Further verification of successful conjugation was observed in a change in negative surface charge with zeta potential increasing from −27 ± 2 mV to −15 ± 3 mV, as seen in Figure 1C,D. The SPR property was also used to indicate successful conjugation and a red shift was observed from 530 nm to 536 nm. Furthermore, T2 MR measurements showed an expected increase in the spin–spin MR from 112 ms for the unbound MPnS to 119 ms for the bound MPnS-mAb, indicating successful conjugation (Figure 1E,F).

### 3.3. Sensitive Detection of E. coli O157:H7 in Aqueous Buffer Solution

**Figure 2:**
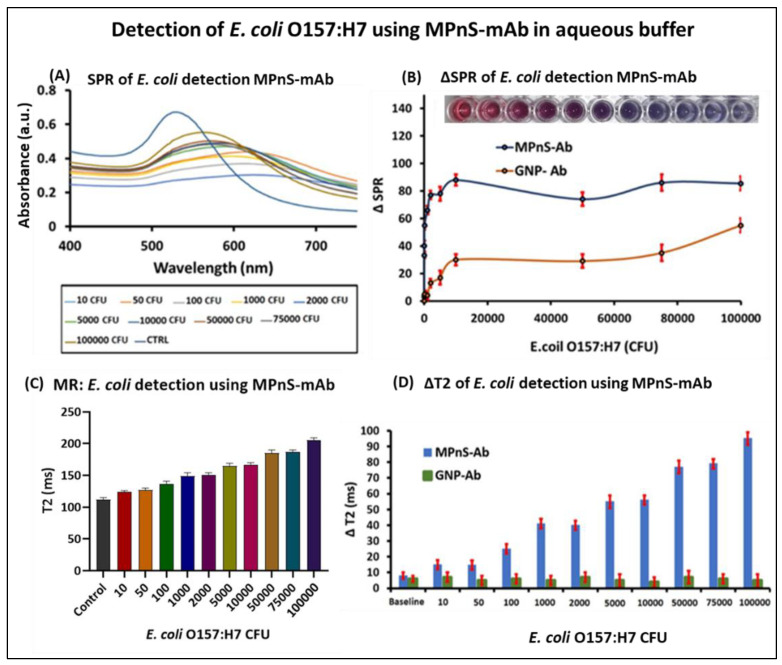
Multimodal detection of *E. coli* O157:H7: (**A**) Increasing CFUs of *E. coli* O157:H7 (10^1^ to 10^5^) were added to the functional MPnS-mAb and the corresponding UV-Vis spectra showing detection. (**B**) Change in absorption maxima (ΔSPR) of MPnS-mAb conjugate and that compared with GNPs-mAb, inset: color changes in response to different CFUs of *E. coli* O157:H7 mixed with MPnS-mAb. (**C**) Corresponding T2 MR values of detection. (**D**) ΔT2 values compared with detection using GNPs-mAb.

Based on the unique plasmonic and MR properties of MPnS, we designed a biosensing assay for the detection of *E. coli* O157:H7, achieving higher sensitivity through spin–spin magnetic relaxation and visual detection based on plasmonic variations. The choice of the target pathogen was guided by the fact that it has an infective cell count of 10 to 100 viable cells and, therefore, it is vital to detect the trace amounts of this pathogen in a rapid and sensitive fashion. The assay procedure involved incubating MPnS-mAb (1.0 mM) with increasing CFUs of *E. coli* O157:H7 spiked in 1X PBS solution (pH 7.4) for 30 min at 25 °C in a 96-well plate. As shown in Figure 2A, a visual change in SPR was observed with the increasing target. This red shift is attributed to the enhanced binding between the target and MPnS-mAb with subsequent aggregation of MPnS, thus confirming the presence of GNPs within the composite nanosensors. This effect was further realized by visual color changes within a few minutes from ruby-red to purple solutions that can be observed with the naked eye (inset, Figure 2B). In contrast, in the control sample (first well) with buffer only, no visible color change was observed, indicating unaggregated MPnS in the solution. A quantitative evaluation of CFU counts was conducted by monitoring the corresponding SPR shifts (∆SPR, Figure 2B) in UV-Vis measurements. The wavelength maximum shift showed an incremental dose-dependent response with increasing CFUs in early-stage infections (10^1^–10^3^). This aggregation-based assay leverages localized surface plasmon resonance changes showing a narrow linear range of detection; however, it was sensitive enough to detect as low as 10 CFUs. In addition, SPR and T2 MR experiments were performed for the detection using functional GNPs-mAb only (Figure S3). By comparing the detection ∆SPR plots of MPnS-mAb and GNPs-mAb under identical conditions, it was observed that MPnS displayed higher sensitivity with better plasmonic enhancement. The MR values of the same samples showed a positive correlation between T2 MR and increasing CFUs of target bacteria (Figure 2C). This indicates that, for the high sensitivity in detection as bacterial CFU increases, it binds and clusters MPnS, thereby replacing neighboring water molecules. As anticipated, minimal ∆T2 was observed in the control sample and with non-magnetic GNPs only (Figure S3). The ∆T2 MR measurements of MPnS (Figure 2D) indicated that a concentration lower than 10 CFUs can be detected within a few minutes. The linear dynamic range of the assay obtained from T2 results was high, between 10^1^ and 10^5^ CFUs. In addition, the assay procedure is simple without requiring additional sample preparation and enrichment steps. Notably, this is an important illustration of achieving improved detection sensitivity due to the synergistic effects of nanomaterials with different characteristic properties.

### 3.4. Specificity in the Detection of E. coli O157:H7

**Figure 3:**
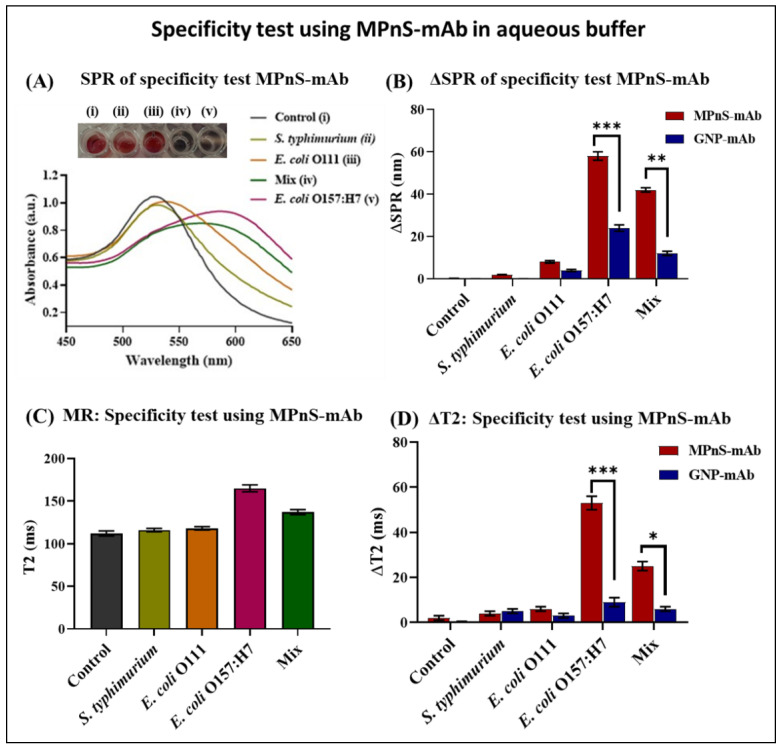
Specificity of antibody-conjugated MPnS in the presence of other bacterial cross-contaminants and compared with functional GNPs: (**A**) Specificity test was conducted in the presence of non-target pathogens by collecting UV-Vis data and colorimetric readout, and (**B**) compared with GNPs-mAb for target bacteria *E. coli* O157:H7 detection. In (**C**,**D**), additional specificity testing was performed by monitoring changes in T2 MR values. Statistically significant differences (*p* < 0.001 or ***, 0.01 or **, and 0.1 or *) were observed when data points for MPnS-mAb and GNP-mAb were compared for *E. coli* O157:H7 and Mix.

To determine the specificity of our MPnS-mAb nanoplatform, we used other common foodborne pathogens including *Salmonella typhimurium* (*S. typhimurium*) as well as isotypic control *E. coli* strain, *E. coli* O111. We also tested the sensitivity of our detection platform with a mixture of all pathogens as real-world samples could potentially be contaminated with an assortment of bacteria. As seen in Figure 3A, SPR absorbance had a marked red shift from 530 nm to 600 nm in the presence of pure *E. coli* O157:H7, with a shift close to 585 nm as expected for a lower bacterial load using a mixture of pathogens rather than a pure sample. Likewise, Figure 3B demonstrates the higher detection sensitivity of MPnS-mAb as compared to using only antibody-conjugated functional GNPs-mAb (Figure S4). As seen, there was a significant difference in the SPR signals between target *E. coli* O157:H7 and the mixture containing *E. coli* O157:H7 compared to other non-target pathogens *E. coli* O111 and *S. typhimurium*, indicative of high specificity in this MPnS-based detection. Next, we tested T2 MR for all samples with results indicating higher specificity of detection for *E. coli* O157:H7 and the target pathogen in the mixed sample as well (Figure 3C). Additionally, ΔT2 results further corroborated our SPR findings, where the T2 MR signal for non-targeted bacteria was comparable to that of the control sample, with a spike in T2 MR measurements noticeably for the target pathogen and for the mixed samples (Figure 3D). These results also indicate the advantage of using MPnS-mAb nanosensors with MR modality when compared to GNP-based detections.

### 3.5. Saturation Studies for MPnS-mAb

**Figure 4:**
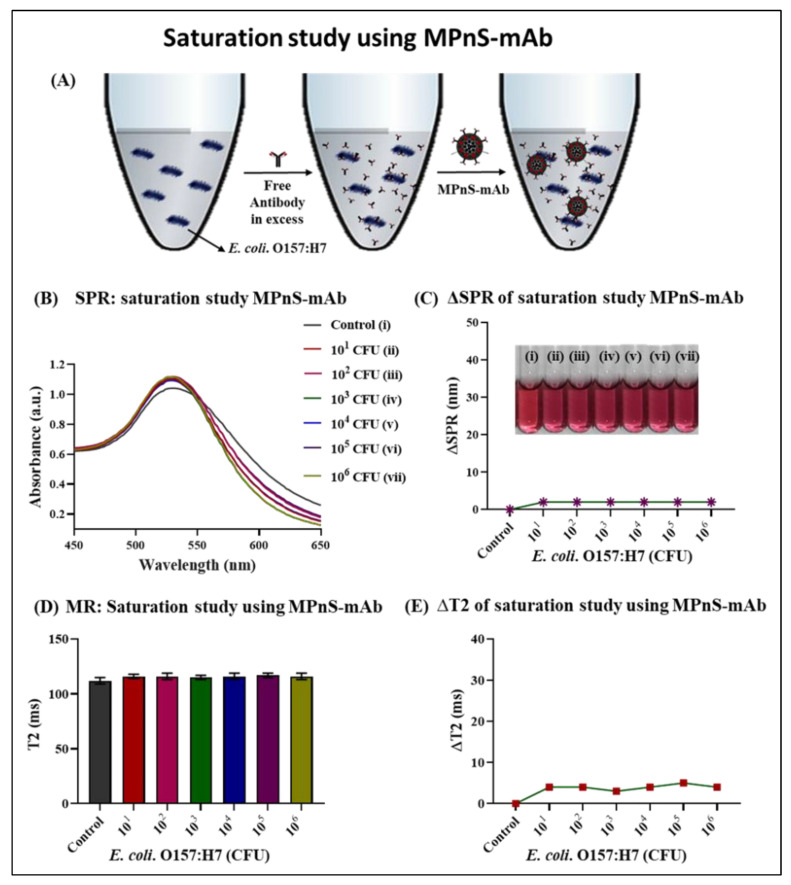
Saturation assay for functional MPnS-mAb: (**A**) Schematic diagram of the assay principle where excess of free *anti*-*E. coli* O157:H7 (10 µM) was incubated with increasing CFUs followed by addition of MPnS-mAb conjugated with *anti*-*E. coli* O157:H7. (**B**) Representative UV-Vis spectra showing no indication of binding or detection, and therefore, (**C**) no changes in absorption maxima (Δ SPR) and color. (**D**) As expected, no sign of binding was observed in T2 MR experiments, and (**E**) displays little to no changes in ΔT2 MR values, further verifying specificity.

To further examine the specificity of our sensing platform and verify that the binding between the *E*. *coli* O157:H7 and MPnS-mAb is due to the presence of targeting antibody, saturation assays were performed. Free *anti-E. coli* O157:H7 IgG (10 µM) was pre-incubated with increasing CFUs of *E. coli* O157:H7, followed by the addition of MPnS-mAb. As shown in Figure 4, the addition of free antibodies resulted in minimal changes in ∆SPR and ∆T2 values. Results indicated that there was no shift in SPR wavelength despite increasing CFUs of *E. coli* O157:H7 (Figure 4B,C). This was due to the fact that the pathogen bacteria were saturated with an excess of free *anti-E. coli* O157:H7 antibodies and thereby, unable to aggregate on the surface of MPnS-mAb. This observation was also reflected as there was no visual change in the color of MPnS-mAb after mixing, indicative of the lack of aggregation (inset, Figure 4C). The antibody-specific binding and the pathogen detection were further supported by T2 MR experiments. The data showed (Figure 4D,E) the absence of any noticeable change in spin–spin magnetic relaxation, further indicating no displacement of neighboring water molecules due to the inability of MPnS-mAb to bind to the target pathogen. These results indicated the ability of our functional MPnS to detect a specific pathogen from an environmental sample.

### 3.6. Sensitive and Specific Detection of E. coli O157:H7 in Complex Media

**Figure 5:**
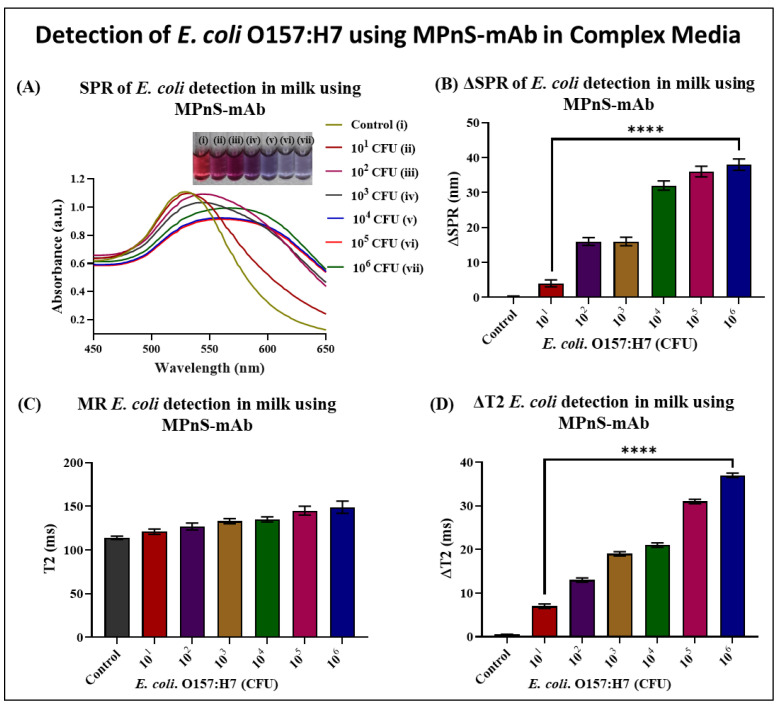
Evaluation of analytical sensitivity in complex media: (**A**,**B**) UV-Vis measurements, and (**C**,**D**) T2 MR experiments indicated the ability of *E. coli* O157:H7 detection in spiked milk. Statistically significant differences (*p* < 0.0001 or ****) were observed when ΔSPR and ΔT2 data points in milk were compared at CFUs 10^1^ and 10^6^.

Following primary detection assays in a simple buffer, the analytical performance of MPnS was evaluated in complex media such as milk. The assay procedure involved spiking milk with increasing *E. coli* O157:H7 CFU concentrations before incubating with MPnS-mAb (1.0 mM), followed by detection using SPR, T2 MR, and colorimetry modalities. The color transition from ruby-red to purple was more markedly noticed around 10^3^ CFUs with a drastic change at 10^4^ CFUs, which was also reflected in the SPR analysis with the red shift in wavelength from around 530 nm to around 575 nm (Figure 5A). This was also reflected in Figure 5B, where the most noticeable ΔSPR increase was around 17 nm for 10^2^ CFUs and then another spiked increase around 32 nm at 10^4^ CFUs. Based on SPR and colorimetric measurements, it was observed that the detection sensitivity was compromised in complex media. The T2 MR experiments showed a steady increase as bacterial concentrations increased (Figure 5C,D), however, no noticeable changes in detection sensitivity in ΔT2 values were seen in complex media compared to simple buffer. This is expected since the MR technique is independent of light, and the detection assay performance is similar in suspensions as well as in heterogeneous food samples.

The specificity of our MPnS-mAb was further tested in spiked milk samples using *S. typhimurium*, *E. coli* O111, *E. coli* O157:H7, and finally spiked with a mixture of all three pathogens (Figure S5). UV-Vis experiments showed no change in SPR peaks with no visible color change in the presence of *S. typhimurium* and *E. coli* O111, indicating the absence of any major cross-reactivity. We also evaluated the specificity by including a sample featuring a mixture of pathogens. As expected, the mixture demonstrated reduced *E. coli* O157:H7 detection sensitivity, however, the specificity towards the target pathogen was not compromised. Similarly, the detection specificity was tested in milk using the T2 MR modality, which showed similar results in detection specificity (Figure S5). While not large increases, the T2 MR values of *E. coli* O157:H7 (165 ms) and mixed pathogens (140 ms) detections distinguished well from the control and non-target bacteria samples (around 110 ms). These promising results in complex media indicated that our MPnS-mAb could be used as an inexpensive, highly sensitive, and specific assay in the agrifood industry to help prevent outbreaks of *E. coli* O157:H7.

## 4. Conclusions

In summary, this work offers a new strategy for synthesizing magneto-plasmonic nanosensors (MPnS) with integrated magnetic relaxation (T2 MR), plasmonic (SPR), and colorimetric properties. MPnS was engineered by encapsulating several GNPs within the polymeric coating of IONPs, which resulted in amplified signal generation due to enhanced optical properties. Moreover, the combination of different characteristics of nanomaterials in a single nanosensor platform allowed for ultrasensitive detection and at the same time is simple to be adopted in field settings.

In our studies using *E. coli* O157:H7 as a target pathogen, multiparametric readouts including ∆SPR, ∆T2, and colorimetric detection were presented in a simple buffer. The sensitive MR modality in MPnS allowed the detection of bacterial concentration within 10^1^ CFU counts and offered a large dynamic range of detection from 10^1^ to 10^5^ with low turnaround times. The limit of detection achieved using MPnS is comparable to other ultrasensitive assays, however, without requiring extra sample processing and enrichment steps. As illustrated in our results, the successful merging of plasmonic and magnetic properties also enhanced the optical properties of MPnS that allowed detectable color changes at lower CFU counts compared to conventional GNP-based assays. It is worth emphasizing that the MPnS-based assay is highly specific as no cross-reactivity was detected with the tested pathogens. Assays conducted in simple and complex matrices indicated comparable T2 signals, however, lower bacterial counts were detected using SPR and colorimetric readouts. Low analytical recoveries of *E. coli* O157:H7 were ascribed to the influence of background signal from milk that affected the signal generation. Overall, our proposed platform has all the features for achieving low detection limits for *E. coli* O157:H7 while retaining simplicity for field settings. It could be an inexpensive and easy-to-use assay with high specificity and sensitivity in the detection of *E. coli* O157:H7 both in the US and other countries confronting this global health issue.

## Data Availability

Not applicable.

## Supplementary Materials

The Supporting Information is available at https://www.mdpi.com/article/10.3390/bios13010109/s1. Scheme S1. Schematic presentations of the syntheses of (A) gold nanoparticles (GNPs) and (B) iron oxide nanoparticles (IONPs); Figure S1. Characterization studies of IONPs and GNPs. (A) Average diameters of GNPs and IONPs, (B) zeta potentials of GNPs and IONPs, (C) UV-Vis absorption spectra (SPR) of GNPs and IONPs, inset: corresponding images of (i) GNPs and (ii) IONPs solutions. (D) T2 values of the nanoparticles, indicating GNPs are non-magnetic; Figure S2. Spin-spin magnetic relaxation (T2 MR) plots of (A) IONPs, (B) GNPs, (C,D) MPnS and its conjugate. Inset showing the color of original nanoparticles; Figure S3. Detection of *E. coli* O157:H7 using GNPs-mAb. Increasing CFUs of *E. coli* O157:H7 was added using antibody functionalized GNPs. (A) Representative UV-Vis spectra at different CFU concentrations of target pathogen. (B) ΔSPR changes and colorimetric readout in response to different CFUs. (C) T2 values and corresponding (D) ΔT2 values for the pathogen detection, indicating MR modality is not applicable for GNPs-based detections; Figure S4. Determination of *E. coli* O157:H7 detection specificity of antibody conjugated GNPs in simple buffer in the presence of other bacterial cross-contaminants. (A,B) UV-Vis measurements and color changes indicative of detection, which showed in the changes in absorption maxima ΔSPR. (C) T2 values and (D) and corresponding ΔT2 were determined for different CFUs of target bacteria in simple buffer. No conclusive results obtained using magnetic relaxometer; Figure S5. Determination of detection specificity of MPnS-mAb in complex media in the presence of other bacterial cross-contaminants. (A) UV-Vis measurements and color changes, and (B) changes in absorption maxima (ΔSPR) indicated specificity in detection was not compromised in complex media. (C,D) T2 MR values and corresponding ΔT2 values were determined for different CFUs of target bacteria spiked in milk, further indicated magnetic relaxation properties of MPnS is independent of media turbidity; Table S1. Specific detection of *E. coli* O147:H7. Table S1. Specific detection of *Escherichia coli* O157:H7 using different sensing platforms.
