# Supplementary material for: Tunable Magneto-Plasmonic Nanosensor for Sensitive Detection of Foodborne Pathogens
Source: Biosensors (Basel). 2023 Jan 7;13(1):109. doi: 10.3390/bios13010109 (PMC9856065; doi:10.3390/bios13010109)
Supplement: Supplementary file 1 [file biosensors-13-00109-s001.zip › biosensors-2059964-supplementary.pdf]

Tunable Magneto-Plasmonic Nanosensor For Sensitive Detection of Foodborne Pathogens

Tuhina Banerjee [2,*], Nilamben Panchal [1], Carissa Sutton [2], Rebekah Elliott [1], Truptiben Patel [1], Kajal Kajal [1], Eniola Arogunyo [1], Neelima Koti [1] and Santimukul Santra [1,*]

1 Department of Chemistry, College and Arts and Sciences, Pittsburg State University, 1701 S. Broadway Street, Pittsburg, KS 66762, USA
2 Department of Chemistry and Biochemistry, College of Natural and Applied Sciences, Missouri State University, 901 S. National Avenue, Springfield, MO 65897, USA
* Correspondence: tbanerjee@missouristate.edu (T.B.); ssantra@pittstate.edu (S.S.)

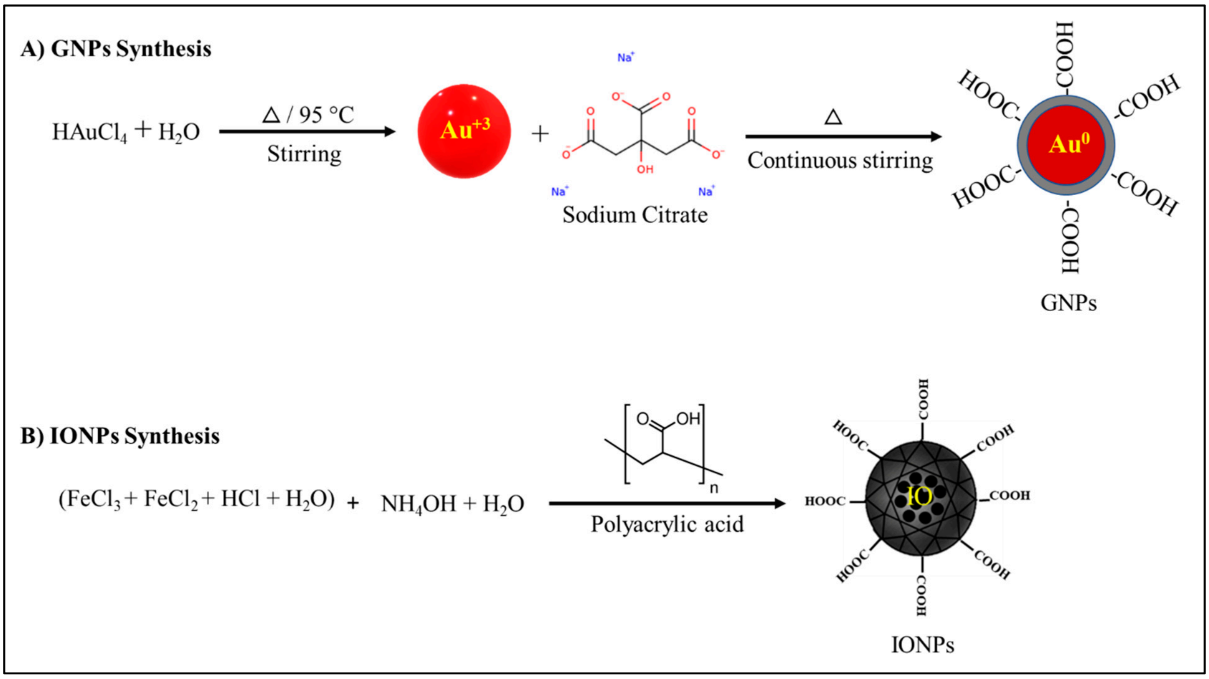

Scheme S1. Schematic presentations of the syntheses of **A**) gold nanoparticles (GNPs) and **B**) iron oxide nanoparticles (IONPs).

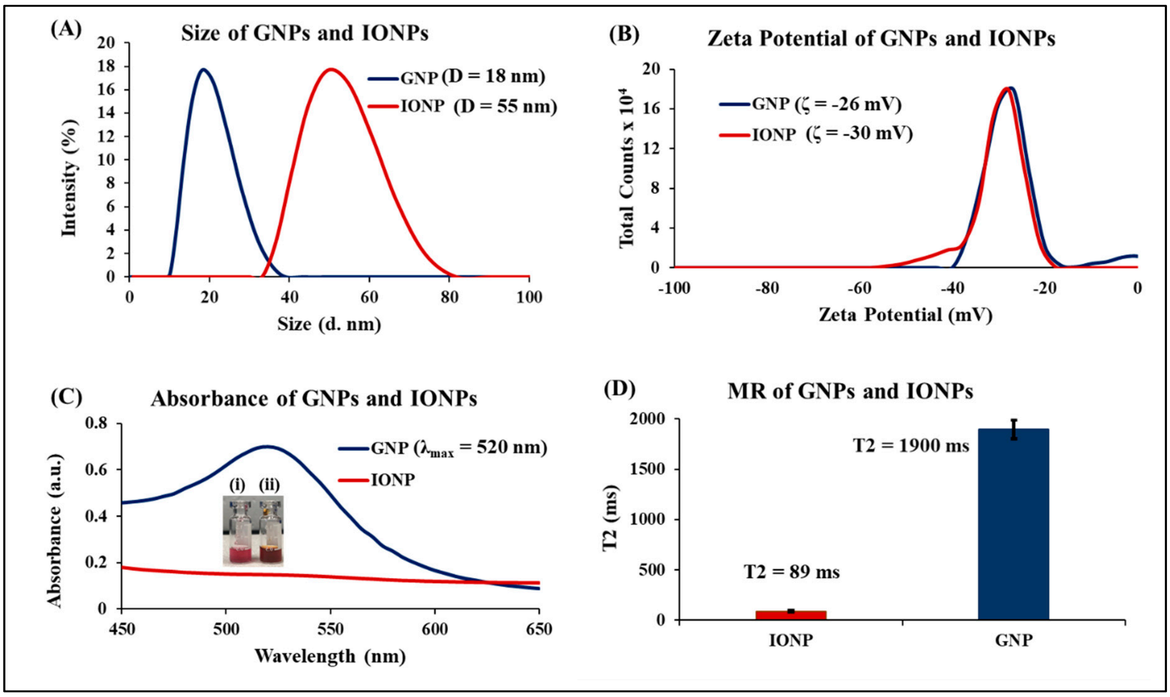

Figure S1. Characterization studies of IONPs and GNPs. (**A**) Average diameters of GNPs and IONPs, (**B**) zeta potentials of GNPs and IONPs, (**C**) UV-Vis absorption spectra (SPR) of GNPs and IONPs, inset: corresponding images of (**i**) GNPs and (**ii**) IONPs solutions. (**D**) T2 values of the nanoparticles, indicating GNPs are non-magnetic.

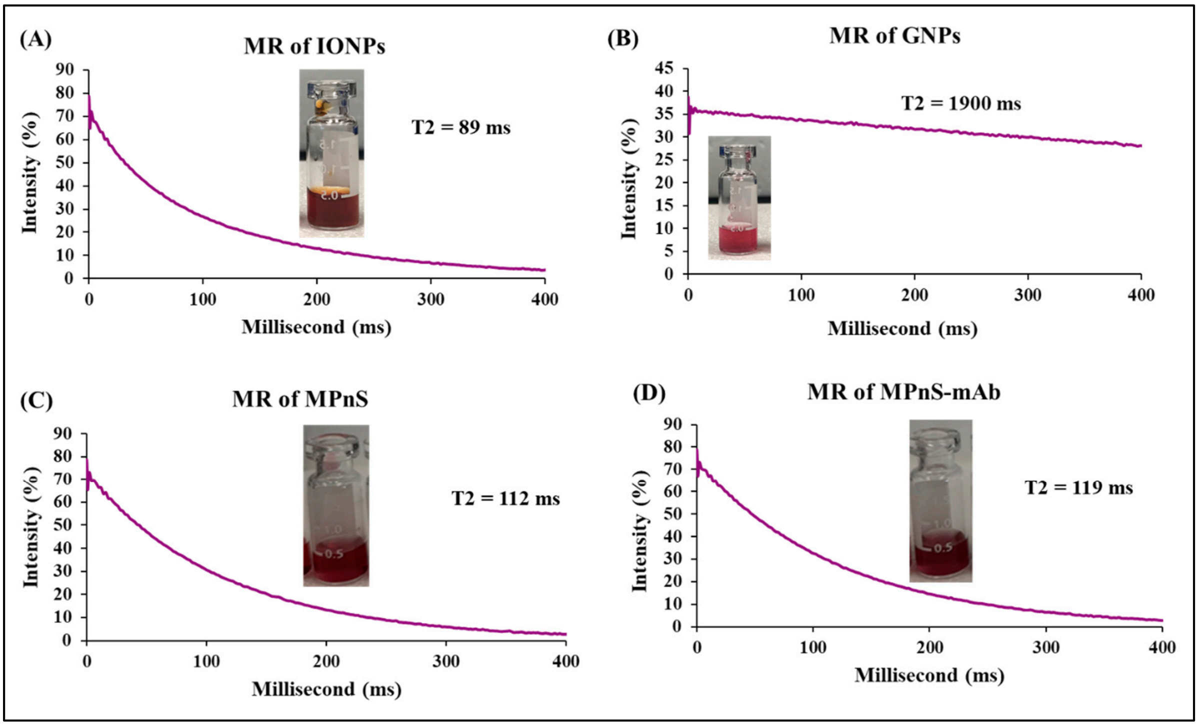

Figure S2. Spin-spin magnetic relaxation (T2 MR) plots of of (**A**) IONPs, (B) GNPs, (**C** and **D**) MPnS and its conjugate. Inset showing the color of original nanoparticles.

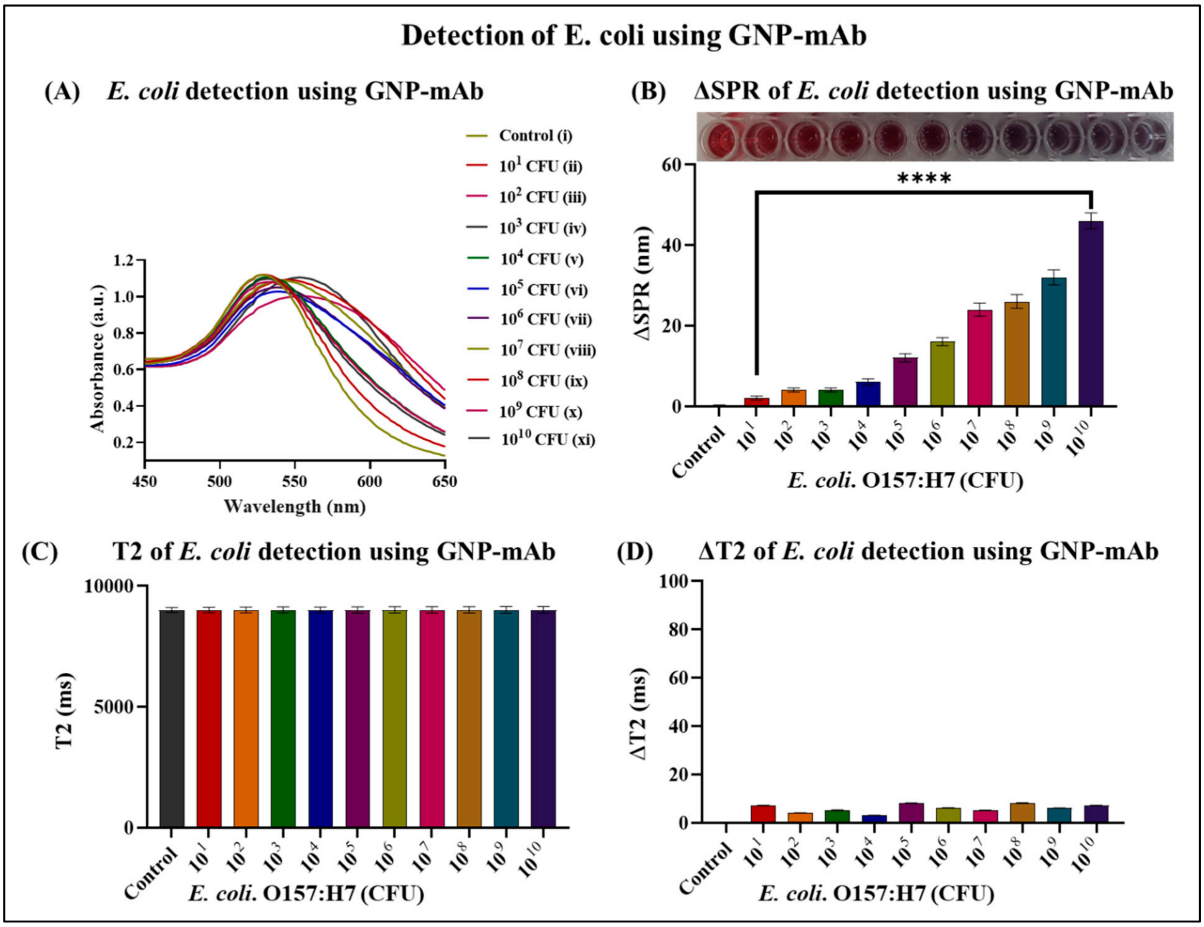

Figure S3. Detection of *E. coli* O157:H7 using GNPs-mAb. Increasing CFUs of *E. coli* O157:H7 was added using antibody functionalized GNPs. (**A**) Representative UV-Vis spectra at different CFU concentrations of target pathogen. (**B**) ΔSPR changes and colorimetric readout in response to different CFUs. (**C**) T2 values and corresponding (**D**) ΔT2 values for the pathogen detection, indicating MR modality is not applicable for GNPs-based detections.

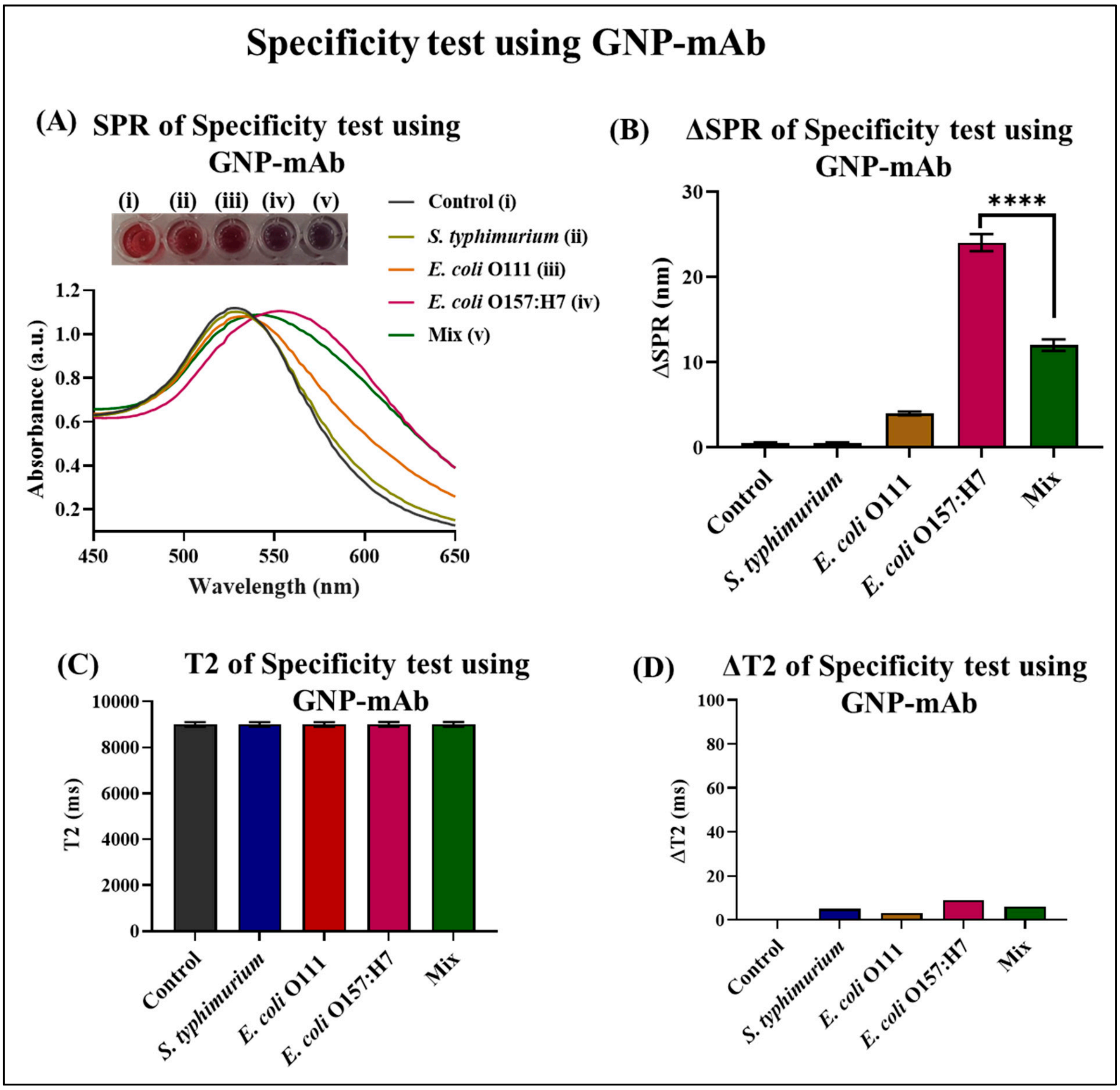

Figure S4. Determination of *E. coli* O157:H7 detection specificity of antibody conjugated GNPs in simple buffer in the presence of other bacterial cross-contaminants. (**A-B**) UV-Vis measurements and color changes indicative of detection, which showed in the changes in absorption maxima ΔSPR. **(C)** T2 values and **(D)** and corresponding ΔT2 were determined for different CFUs of target bacteria in simple buffer. No conclusive results obtained using magnetic relaxometer.

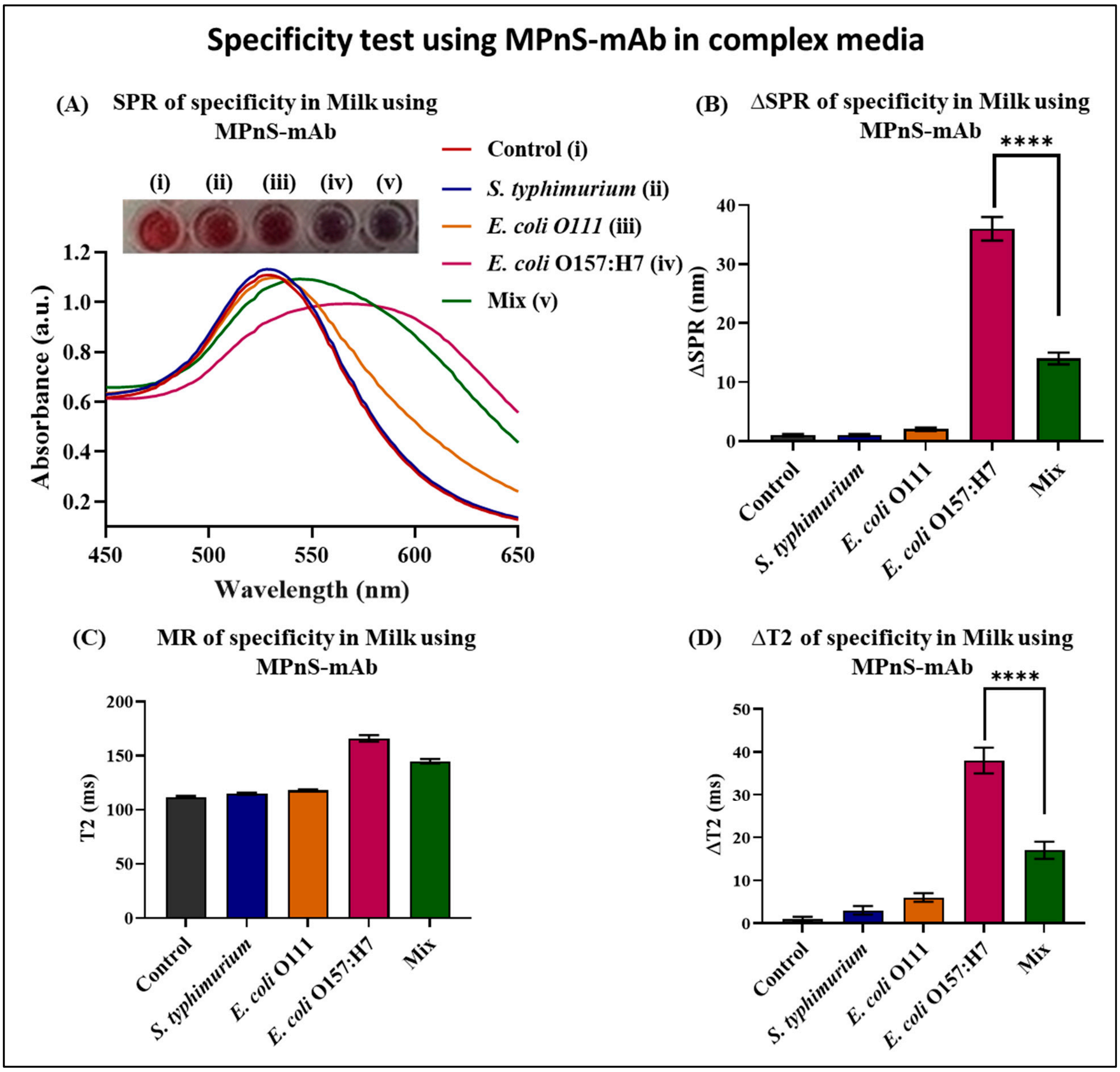

Figure S5. Determination of detection specificity of MPnS-mAb in complex media in the presence of other bacterial cross-contaminants. (**A**) UV-Vis measurements and color changes, and (**B**) changes in absorption maxima (ΔSPR) indicated specificity in detection was not compromised in complex media. (**C** and **D**) T2 MR values and corresponding ΔT2 values were determined for different CFUs of target bacteria spiked in milk, further indicated magnetic relaxation properties of MPnS is independent of media turbidity.

Sensor Type	LOD (CFUs/mL)	Ref
AuNP-Immunochromatographic assay	12.5	60
ELISA	1×10^4	61
Chemiluminescence biosensor	130	62
Fluorescent microsphere-based Immunochromatographic assay	3×10^5	63
Microfluidic Biosensor	10	64
Polydopamine-NP-assisted polymerase chain reaction	6.7×10^4	65
Gold-Shell Silica-Core Nanospheres	100	66

Table S1. Specific detection of *Escherichia coli* O157:H7 using different sensing platforms.
